# Strengthening nursing knowledge and skills in perioperative cleft care: a focused training approach in Nigeria’s surgical healthcare plan

**DOI:** 10.3389/fmed.2025.1502456

**Published:** 2025-03-10

**Authors:** Olubunmi Aiyedun Lawal, Ngozika Opara, Chisom Rachel Udeigwe-Okeke, Aderonke Omoyeni Obisesan, Justina Seyi-Olajide, Oti Nimi Aria, Mohammed Adam Sheikh Abdullahi, Nkeiruka Obi, Emmanuel A. Ameh

**Affiliations:** ^1^March Healthcare Initiative, Abuja, Nigeria; ^2^NSOANP-Smile Train Technical Review Team, Abuja, Nigeria; ^3^Division of Paediatric Surgery, Department of Surgery, National Hospital, Abuja, Nigeria; ^4^Department of Anaesthesia, National Hospital, Abuja, Nigeria; ^5^Department of Surgery, Lagos University Teaching Hospital, Lagos, Nigeria; ^6^NSOANP Implementation Committee, Federal Ministry of Health, Abuja, Nigeria; ^7^Department of Surgery, Rivers State Teaching Hospital, Port Harcourt, Nigeria; ^8^Department of Oral and Maxillofacial Surgery, University of Maiduguri Teaching Hospital, Maiduguri, Nigeria; ^9^Smile Train, Africa Office, Lagos, Nigeria

**Keywords:** surgery, safety, outcomes, perioperative, nursing, training

## Abstract

**Background:**

Safe perioperative nursing care is crucial to improving outcomes of surgical care. This is a report on the pilot implementation of a nursing training programme aimed at strengthening safe perioperative nursing care in Nigeria, aligning with the nation’s strategic framework for surgical, obstetric, anaesthesia, and nursing plan. The aim of this report is to highlight the need to incorporate perioperative nursing training into efforts to scale up access to surgical care in low resource settings.

**Methods:**

The Nursing Care Saves Lives (NCSL) training programme which was designed for training in perioperative nursing of cleft lip and palate patients, was adapted for perioperative nursing training. A 5-day intensive training was deployed, involving lectures, hands-on activities, simulations, and group problem-solving exercises. Pre- and post-training surveys were administered, and participant feedback and 3-months follow-up assessments obtained. The data has been analysed using descriptive statistics.

**Results:**

Twenty-six participants who were nurses involved in perioperative care, from both public and private hospitals, completed the training. Pre-training evaluation scores ranged from 23 to 72% (median 68%), increasing significantly to 61–98% (median 76%) post-training (*p* = 0.0001). Participants rated all training contents as useful, with high satisfaction in neonatal resuscitation and basic life support skills. Infection prevention and control, helping babies breathe, and effective communication were identified as key learnings. Recommendations for future training included facilitation skills, nutrition, and research. Although 10 (40%) participants organised step down trainings, limited funding and training materials were key barriers to step down.

**Conclusion:**

The NCSL training programme has the potential for promoting and strengthening safe perioperative nursing care. Strategic efforts are needed to scale up and expand access to this training within the wider perioperative nursing community, to enhance patient safety and surgical outcomes in the setting.

## Introduction

In Nigeria, ensuring safe perioperative nursing care remains a critical priority within the broader context of advancing healthcare delivery. Nursing training in the country is regulated by the Nursing and Midwifery Council of Nigeria (NMCN), with perioperative nursing offered as a post-basic specialization. However, there is a shortage of trained perioperative nurses, particularly in district and general hospitals. Most nurses working at these hospitals have not gone through the post-basic specialisation in perioperative nursing. In addition, paediatric perioperative nursing is not well integrated into the training of these nurses. Further, access to hands-on training, simulation, and continuous professional development in these aspects are limited.

There are important gaps in paediatric perioperative nursing care in the setting. Many nurses lack specialized training in recognizing early post-surgical complications, and standards of care protocols and checklists are not usually in use outside tertiary hospitals. It has been shown that continuing nursing education in low- and middle-income countries is effective in improving their knowledge, and hence patient outcomes and quality of care (1).

Recognizing the pivotal role of nursing in perioperative settings, the implementation of comprehensive training programmes becomes imperative to enhance positive patient outcomes and mitigate risks associated with surgical interventions. Moreover, the outcome of cleft surgery depends on both surgical expertise and quality of postoperative nursing care. Inadequate perioperative nursing care can lead to airway problems and wound complications following cleft surgery.

The Nursing Care Saves Lives (NCSL) training programme is a meticulously designed curriculum crafted to improve the safety and effectiveness of post-operative nursing care for children following cleft surgery (2). Rooted in evidence-based practices and tailored to local contexts, this programme is a holistic approach encompassing theoretical knowledge, practical skills development, and attitudinal enhancements necessary for delivering optimal perioperative care. The course has already been deployed by Smile Train but being piloted by Nigeria’s NSOANP. There is presently no published data on previous pilots and evaluation of the programme.

This manuscript presents a report on the pilot implementation of the nursing training programme tailored to strengthen safe perioperative care in Nigeria. This aligns with the nation’s strategic framework for surgical, obstetric, anaesthesia, and nursing services (3). The report is intended to highlight the feasibility and potential impact of this training and application to similar settings.

## Methods

Nursing Care Saves Lives (NCSL) is a 5-day training programme designed by Smile Train (a cleft lip and palate focused organisation that supports the delivery of free surgery to cleft patients) provides nurses with essential skills needed to safely administer pre- and post-operative nursing care for patients with cleft lip and palate. The curriculum involves lectures and didactic instruction, while also incorporating interaction between trainer and trainees. Participants practice essential skills such as infant resuscitation, patient monitoring, skill-building exercises, simulation of scenarios, and group problem solving centred on four pillars of patient assessment, post-operative nursing care, postoperative complications, and post-operative nursing interventions. Particular attention is given to early recognition of post-operative complications and essential nurse-initiated actions. The existing NCSL curriculum (2) was slightly modified to include helping babies breathe (HBB), pulse oximetry, cleft nutrition, and infection prevention and control. Although the NCSL was designed for cleft care, it was adapted to apply to safe perioperative nursing care in general to meet local needs.

### Training objectives

**Figure 1:**
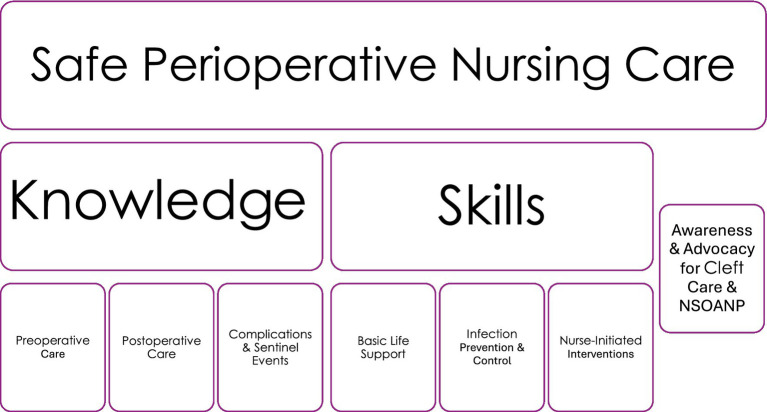
Training objectives to strengthen safe perioperative nursing care. NSOANP, National Surgical, Obstetrics, Anaesthesia and Nursing Care for Nigeria.

The training was done as part of the ongoing implementation of the national surgical, obstetrics, anaesthesia, and nursing plan (NSOANP) in alignment with one of the key objectives of strengthening perioperative nursing care in Nigeria (Figure 1).

### Participants

Four participants each from the 6 geopolitical zones of Nigeria, and 2 from the federal capital territory were selected for the training based on their performance in a web-based quiz. The aim of the quiz was to select 25 participants as recommended by the NCSL curriculum, to ensure hands-on experience and involvement of all participants (2). A total of 26 participants were eventually selected for the training. The selected participants were registered nurses involved in perioperative care.

### Deployment of training

The training took place in August 2021. This was a 5-day, intensive training attended by the 26 previously selected participants. Activities carried out to ensure effective learning experiences and skills acquisition, included:

1. Team quizzes.
2. Group-based care planning.
3. Problem-solving and group feedback.
4. Hands-on activities using medium fidelity feedback mannequins.
5. Action planning using flip charts and wall charts.

### Training evaluation

Pre- and post- training evaluations were administered to the participants to test their knowledge before and after the training. In addition, post training feedback survey was administered to the participants using a 5-point Likert scale. The participants were also followed up for 3 months to evaluate the early impact of the training. Data for the follow up was on step down training of others and was collected using the WhatsApp group of the participants.

Continuing professional development points were awarded by the Nursing and Midwifery Council of Nigeria (NMCN) as part of the mandatory continuing professional development programmes to enhance effective learning and a prerequisite for nursing practicing license in the country.

### Data analysis

One of the 26 participants did not complete the pre- and post- training evaluations and has been excluded from analysis. Data from the training have been analysed using Excel Analyse-it^(R)^ statistical software and results presented as descriptive statistics. The 5-point (very useful, useful, neutral, less useful, not useful) Likert scale was collapsed to 3-point scale (useful, neutral, not useful) to facilitate analysis. The difference between the median pre-training and post-training evaluations scores was compared using the Wilcoxon sign rank test and level of statistical significance set at *p* = 0.05.

## Results

There were 15 (60%) females and 10 (40%) males from 9 public hospitals and one private hospital.

### Participant performance

The pre-training evaluation score was 23–72% (median 68%) and post-training evaluation score 61–98% (median 76%), the difference between the two median scores was statistically significant (*p* = 0.0001).

### Participants’ feedback

**Figure 2:**
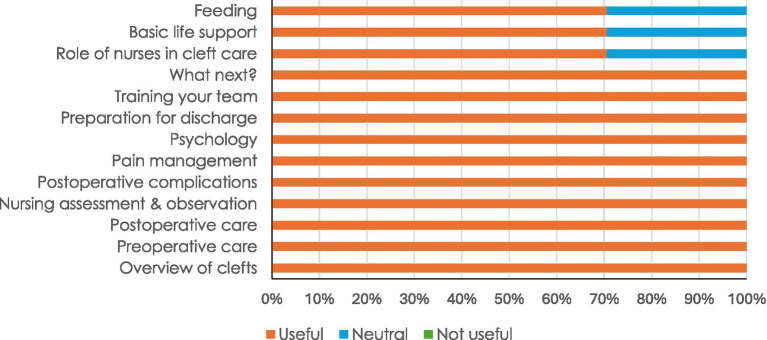
Feedback from 25 participants on usefulness of training content.

Nearly all participants (96 – 100%) rated all contents of the training as useful (Figure 2). Specifically, 10 (40%) participants rated helping babies breathe as what they liked most about the training, 9 (36%) basic life support skills, 7 (28%) infection prevention and control, and 4 (16%) all contents (Table 1). Twenty-four (96%) participants indicated that there was nothing they liked least while one did not respond.

**Table 1:** Participants’ feedback on most liked content and most important thing learnt.

Content	No. (%) *n* = 25
Most liked content	
Helping babies breathe	10 (40)
Basic life support	9 (36)
Infection prevention & control	7 (28)
All contents	4 (16)
Documentation	2 (8)
Nursing assessment & observation	2 (8)
Nursing management of child with cleft	2 (8)
Role of nurse in cleft care	1 (4)
Pre- & post-operative care	1 (4)
Management of emergency situation	1 (4)
Assisted breathing	1 (4)
Most important thing learnt	
Infection prevention & control	11 (44)
Helping babies breathe	10 (40)
Effective communication	8 (32)
Basic life support	5 (20)
Perioperative care of cleft patients	4 (16)
Nursing care of patients with cleft	2 (8)
Overview of clefts	1 (4)
Monitoring of vital signs	1 (4)
Taking part in step down training	1 (4)
Identification of emergencies & quick response	1 (4)
Postoperative care of cleft patients	1 (4)
Feeding	1 (4)
Nutrition	1 (4)

Infection prevention and control (44%), helping babies breathe (40%), and effective communication (32%) were rated as the top three most important things learnt (Table 1). Participants recommended facilitation skills (skills on how to facilitate trainings) (20%), nutrition (16%) and research skills (8%) as the top 3 aspects to be included in future trainings (Table 2).

**Table 2:** Participant’s recommendations on topics to include in future trainings.

Recommended topic	No. (%) *n* = 25
Facilitation skills	5 (20)
Nutrition	4 (16)
Research skills	2 (8)
Needed collaboration	1 (4)
Fluid management	1 (4)
Psychosocial & community engagement	1 (4)
Management of clefts with other congenital conditions	1 (4)
List of designated health facilities and their contacts	1 (4)
Management of perineum during pregnancy	1 (4)

### Step down training

At 3 months of follow up, 10 (40%) of the nurses had organized step down trainings on return to their centres to share their experiences and transfer skills. Lack of funding and training materials were key challenges and barriers against step down training.

## Discussion

In 2015, the world health assembly passed the landmark resolution WHA 68:15 mandating member countries to incorporate emergency and essential surgical and anaesthesia care as integral components of universal health coverage (4). Following this resolution, several countries in sub Saharan Africa, including Nigeria have launched national surgical plans to scale up access to surgical care, while others are working on such plans (5). However, expanding access to surgical care alone will not address the current high morbidity and mortality and poor surgical outcomes in the setting. Such efforts must go hand-in-hand with strengthening the delivery of safe perioperative nursing care. Moreover, nurses form the largest segment of the health workforce in most sub-Saharan Africa countries, including up to 76% in Rwanda (6). Hence, any capacity strengthening programme targeted at nurses has the potential of delivering significant impact on patient outcomes in the setting. In one report using interviews, observations, and pathway maps, and involving 2 upper-middle income, 2 lower-middle income and 1 lower income countries, barriers to safe and effective perioperative care were identified (7). Four key barriers identified in that report included limited human and structural resources, fragmental care pathways, direct and indirect cost of care for patients and patients’ low expectation of care. Addressing limitations in human capacity and patients’ low expectation of care can potentially be mitigated by strengthening the perioperative nursing capacity.

The study measured knowledge acquisition through pre- and post-training evaluations. While confidence in applying knowledge was not explicitly measured, participants’ engagement in hands-on skill-scenarios, problem-solving exercises, and group discussions suggests enhanced procedural confidence. Behavioural change was also evaluated by independent deployment of step-down training sessions within 3 months. Although the study did not directly assess patient outcomes, participants identified infection prevention, neonatal resuscitation, and effective communication as the most impactful components of the training, all of which are critical to improving perioperative nursing care and patient safety.

The findings of this pilot implementation of the perioperative nursing training based on the Nursing Care Saves Lives programme underscore its effectiveness in enhancing perioperative nursing competence and shaping positive attitudes towards patient care.

Participants’ performance, as evaluated through pre- and post-training assessments, demonstrated a significant improvement in knowledge acquisition and retention following the training. The observed increase in median evaluation scores from 68 to 76% post-training highlights the tangible impact of the training in augmenting proficiency in safe perioperative nursing care. Previous reports have emphasised the effectiveness of structured training programmes in enhancing the competencies of healthcare professionals and improving patient outcomes (8, 9). The impact of deploying evidence-based perioperative nursing training programmes has been emphasised (10). In the later report of perioperative nursing training of labour and delivery nurses, it was shown that their knowledge and competence significantly improved after completing training.

The feedback from participants in the present training programme provides some insights into the perceived utility and relevance of its contents. The overwhelmingly positive rating for all training contents is an indication of the comprehensive design of the curriculum and its alignment with perioperative learning needs. Of specific import is the high satisfaction rate with modules addressing neonatal resuscitation (helping babies breathe) and basic life support skills, underscoring the significance of these skills in perioperative nursing care. The important role of effective resuscitation and emergency care training in improving patient survival and reducing perioperative complications has been previously highlighted (10–12).

The participants were registered nurses, already practicing nursing. They were general nurses and paediatric trained nurses. As they were already trained nurses, some level of knowledge was assumed, and their background likely enhanced their knowledge retention and application.

The recommendation of participants regarding topics for inclusion in future trainings, such as skills on how to facilitate trainings, nutrition, and research, offer valuable guidance for curriculum strengthening and programmatic enhancements. Including these topics in future training iterations could further enrich the skills and learning experience for perioperative nurses.

The concept of step-down training, wherein trained nurses disseminate knowledge and skills within their healthcare facilities, holds immense potential for cascading the benefits of the training programme to more nurses and staff. However, limited funding and lack of training materials remain important barriers that require institutional support and resources. In addition, collaboration between these institutions and donors are crucial to the sustainability and scalability of this safe perioperative nursing training across Nigeria.

## Conclusion

The Nursing Care Saves Lives (NCSL) programme employed several approach to assess and track behaviour change among the nurses. Beyond evaluating knowledge acquisition, the programme focused on measuring the application of skills in real-world clinical settings. Structured pre- and post-training evaluations demonstrated significant improvements in clinical decision-making and procedural confidence, indicating both knowledge gain and the ability to translate learning into practice. Simulated scenarios and direct observation during training provided real-time feedback, ensuring skill mastery before participants returned to their clinical roles. Behaviour change was further assessed by monitoring knowledge dissemination, with follow-up showing that 40% of participants had independently conducted step-down training sessions for colleagues within 3 months, reflecting internalisation of key concepts and sustained confidence in their application. In addition, 96–100% of participants rated key components of the training as useful, with infection prevention, neonatal resuscitation, and effective communication identified as the most impactful areas of practice change. The combination of objective skill assessments and subjective self-reports reinforces the programme’s effectiveness in enhancing behavioural change.

The outcome of this pilot implementation has shown that the NCSL training programmes adapted for safe perioperative nursing care in general, has the transformative potential of promoting and strengthening safe perioperative nursing care. The training is desirable in the drive to expand access to timely and safe surgical care in the setting and would enhance patients’ safety and improvements in surgical outcomes. Efforts need to be directed towards refining and scaling up the programme across Nigeria.

## Data Availability

The raw data supporting the conclusions of this article will be made available by the authors, without undue reservation.

## References

[1] Azad A Min J-G Syed S. Sara Anderson - continued nursing education in low-income and middle-income countries: a narrative synthesis: BMJ. Glob Health. (2020) 5:e001981. doi: 10.1136/bmjgh-2019-001981, PMID: 32181001 PMC7042573

[2] Training manual. Nursing care saves lives. New York: Smile Train (2024).

[3] National surgical, obstetric, anaesthesia and nursing plan. (2019). Federal Ministry of Health, Abuja. Available online at: (https://www.pgssc.org/nationalsurgical-planning).

[4] World health Assembly. (2015). Strenghtening emergency and essential surgical care and anaesthesia as a component of universal health coverage. Available online at: (https://apps.who.int/gb/ebwha/pdf_files/WHA68/A68_R15-en.pdf).

[5] Bekele A Alayande BT Powell BL Obi N Seyi-Olajide JO Riviello RR. National surgical healthcare policy development and implementation: where do we stand in Africa? World J Surg. (2023) 47:3020–9. doi: 10.1007/s00268-023-07131-0, PMID: 37550548

[6] Mukantwari J Omondi L Ryamukuru D. Perioperative nursing training in Rwanda in partnership with American universities: the journey so far. Rwanda J Med Health Sci. (2021) 4:185–96. doi: 10.4314/rjmhs.v4i1.13 PMC12257249 40666406

[7] Bedwell GJ Dias P Hahnle L Anaeli A Baker T Beane A. Barriers to quality perioperative care delivery in low- and middle-income countries: a qualitative rapid appraisal study. Anesth Analg. (2022) 135:1217–32. doi: 10.1213/ANE.0000000000006113, PMID: 36005395

[8] Smith AF Youngson GG Jolly BC Green L. Audit of resuscitation skills and knowledge among healthcare providers in a private hospital in Malaysia. Br J Anaesth. (2019) 123:e108–11.

[9] Thomas L Gallauresi B Mehta R. Implementation of an evidence-based pediatric sepsis protocol in a community hospital improves delivery of care. Pediatr Crit Care Med. (2020) 21:e172–80.

[10] Stucky CH Knight AR Dindinger RA Maio S House S Wymer JA. Periop101: improving perioperative nursing knowledge and competence in labour and delivery nurses through an evidence-based education and training program. Mil Med. (2023) 189:24–30. 37956334 10.1093/milmed/usad287

[11] Berglund E Jonsson K Maurex L Anderzén-Carlsson A. Simulation-based team training in a Swedish neonatal intensive care unit: a multidisciplinary approach. BMJ Simul Technol Enhanc Learn. (2018) 4:226–33.

[12] Soar J Monsieurs KG Ballance JHW Barelli A Biarent D Greif R. European resuscitation council guidelines for resuscitation 2015: section 9. Principles of education in resuscitation. Resuscitation. (2015) 95:288–301.26477418

